# Ferromagnetism in ultrathin MoS_2_ nanosheets: from amorphous to crystalline

**DOI:** 10.1186/1556-276X-9-586

**Published:** 2014-10-22

**Authors:** Rongfang Zhang, You Li, Jing Qi, Daqiang Gao

**Affiliations:** 1 Key Laboratory for Magnetism and Magnetic Materials of MOE, Lanzhou University, Lanzhou 730000, People's Republic of China; 2 Gansu Agriculture Technology College, Lanzhou 730000, People's Republic of China

## Abstract

Two-dimensional materials have various applications in the next generation nanodevices because of their easy fabrication and particular properties. In this work, we studied the effects of crystalline order on the magnetic properties of ultrathin MoS_2_ nanosheets. Results indicate that all the fabricated samples show clear room temperature ferromagnetism. The amorphous sample has the larger saturation magnetization than that of the crystallized samples, where the disordered grain boundary or defects in the nanosheets are considered to be responsible for the long-range magnetic order. These MoS_2_ nanosheets with versatile functions may have potential applications in spintronics, nanodevices, and photodevices.

## Background

Magnetic ordering in materials that do not have partially filled *d* or *f* orbitals named defect-induced magnetism (DIM) is a topic of interest in recent years [1-3]. Until now, the DIM phenomenon has been finally observed in a broad spectrum of materials, from several oxides to carbon based materials [4,5] and to the transition-metal dichalcogenide with the atomic thickness [6,7].

Recently, as one of the central members in transition metal dichalcogenide compounds, MoS_2_ with its remarkable electronic properties such as charge density wave transitions in transition metal dichalcogenides make the material interesting from both fundamental and applied research perspectives [8-10]. For example, reports demonstrate strong photoluminescence emergence and anomalous lattice vibrations in single- and few-layered MoS_2_ films [11,12]. Results also indicate that the single-layer MoS_2_ exhibits a high channel mobility (approximately 200 cm^2^V^−1^ s^−1^) and current on/off ratio (1 × 10^8^) when it was used as the channel material in a field-effect transistor [13]. Most recently, it is proposed that the indirect band gap of bulk MoS_2_ with a magnitude of approximately 1.2 eV transforms gradually to a direct band gap of approximately 1.8 eV in single-layer samples [14,15], which is in contrast to pristine graphene with a band gap of approximately 0 eV and few-layered *h*-BN with a band gap of approximately 5.5 eV [16].

Recently, theoretical studies suggested that, although bulk MoS_2_ is a diamagnetic material, it becomes ferromagnetic when MoS_2_ nanoribbons are formed with zigzag edges, defects are induced, or nonmetals such as H, B, C, N, and F are absorbed [17,18]. In addition, formation of magnetic moments was also reported in Mo_n_S_2n_ clusters, nanoparticles, and nanoribbons of MoS_2_ from first principle studies [19-21]. Experimentally, the interesting ferromagnetism phenomenon in nanosheets of MoS_2_ had previously been reported and the observed magnetic signal is attributed to the presence of unsaturated edge atoms [22]. The weak ferromagnetism was also obtained in the freestanding nanosheets and in the bulk MoS_2_ irradiated by proton, which increases the *T*_C_ up to 895 K [23,24]. Further, the existence of magnetism in the pristine MoS_2_ has been reported despite that there are differences depending on the layer thickness [25].

Although such unexpected ferromagnetic behavior ascribed to edge states and defects in MoS_2_ has been reported, there are no experimental observations of ferromagnetism in MoS_2_ with a different degree of crystallization. In this letter, MoS_2_ nanosheets from amorphous to crystalline were prepared by a hydrothermal method reaction at different temperatures. The structure and magnetic properties were studied. Results indicate that all the samples show clear room ferromagnetism and the ferromagnetism weakened with the crystalline order increasing.

## Methods

MoS_2_ nanosheets with different crystalline order were synthesized by the hydrothermal method. Sodium molybdate dihydrate, 1.5 g, and thioacetamide, 2.2 g, were dissolved together in 50 ml of deionized water and stirred for 2 h. Then, the solution was transferred into a Teflon-lined stainless steel autoclave and reacted at different temperatures (180°C, 200°C, 220°C and 240°C) for 24 h. Finally, the products were filtered and washed with water and dried in a vacuum.

X-ray diffraction (XRD, X'Pert PRO PHILIPS with Cu K_α_ radiation) was employed to study the crystal structure. The morphology of the samples was obtained by using the high-resolution transmission electron microscopy (HRTEM, TecnaiTM G2 F30, FEI, Hillsboro, OR, USA). X-ray photoelectron spectroscopy (XPS, Kratos Axis Ultra, Manchester, UK) was utilized to determine the bonding characteristics of the samples. The composition was confirmed by an inductively coupled plasma atomic emission spectrometer (ICP, ER/S). The measurements of magnetic properties were made using the Quantum Design MPMS magnetometer based on superconducting quantum interference device (SQUID).

## Results and discussion

**Figure 1:**
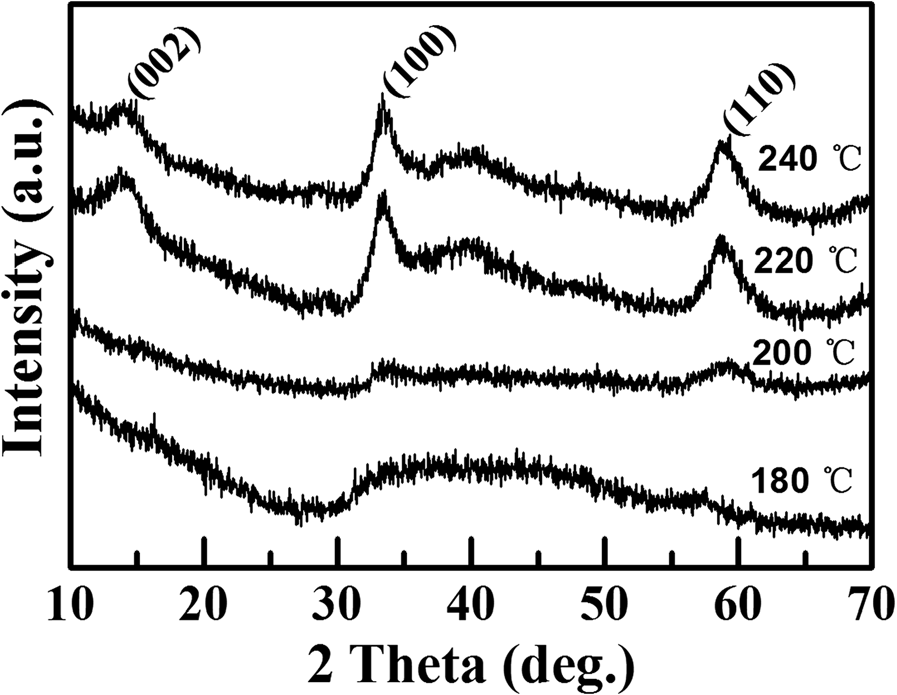
**XRD results for the MoS**_**2 **_**nanosheets synthesized at different temperatures.**

The XRD patterns for the samples are shown in Figure 1. As can be seen that the sample synthesized at 180°C shows only a broad, weak envelope beginning at about 2θ° =30° and continuing out above 60°, the two maxima approximately locate at the (100) and (110) positions of bulk MoS_2_. As the reaction temperature increases, the diffraction peaks appear and increase gradually, indicating that the sample begins to crystallize when the reaction temperature increases to 200°C, while the crystallization order of the sample increases with the reaction temperature further elevating. Comparing all the XRD results, it is clear that pressure (reaction temperature) plays an important role in the crystallization of the MoS_2_.

**Figure 2:**
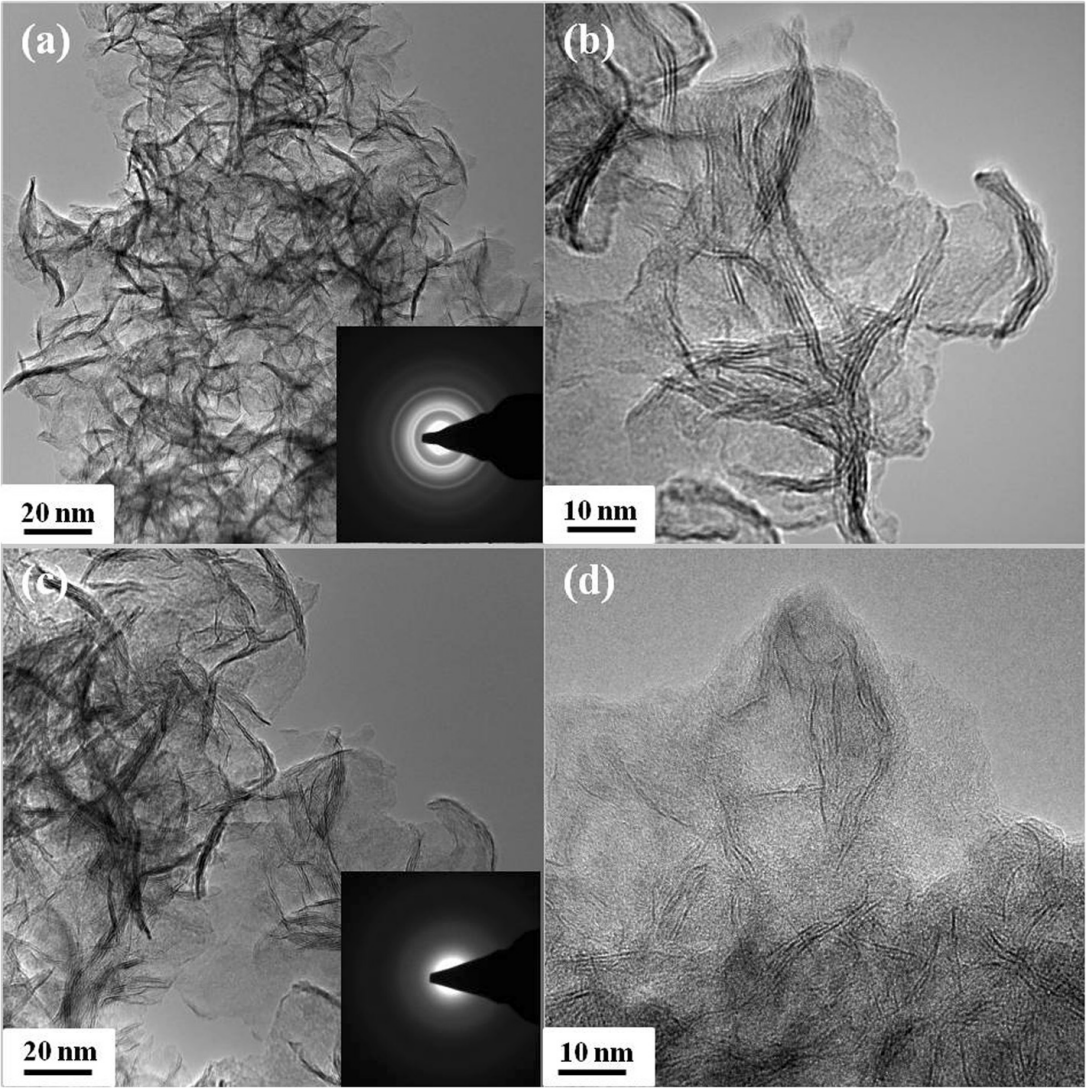
**The TEM results for MoS**_**2 **_**nanosheets synthesized at (a, b) 240°C and (c, d) 180°C.** The inset shows the corresponding selected area electron diffraction (SAED) result.

Further, the morphology of the samples was also captured by the TEM, and the results are shown in Figure 2. It can be seen that all the samples have the sheet-like morphology. The corresponding selected area electron diffraction (SAED) results for the MoS_2_ nanosheets given in inset in Figure 2a,c reveal the crystallization order of the samples, that is, the sample synthesized at 180°C shows the amorphous nature and the sample synthesized at 240°C had a good crystallization, which is consistent with the XRD results. HRTEM investigation in the edge areas was a common and direct method to determine the layer numbers microscopically. In our case, as in Figure 2b, three to four dark and bright patterns can be readily identified, indicating that the sample is stacked with three to four single layers.

**Figure 3:**
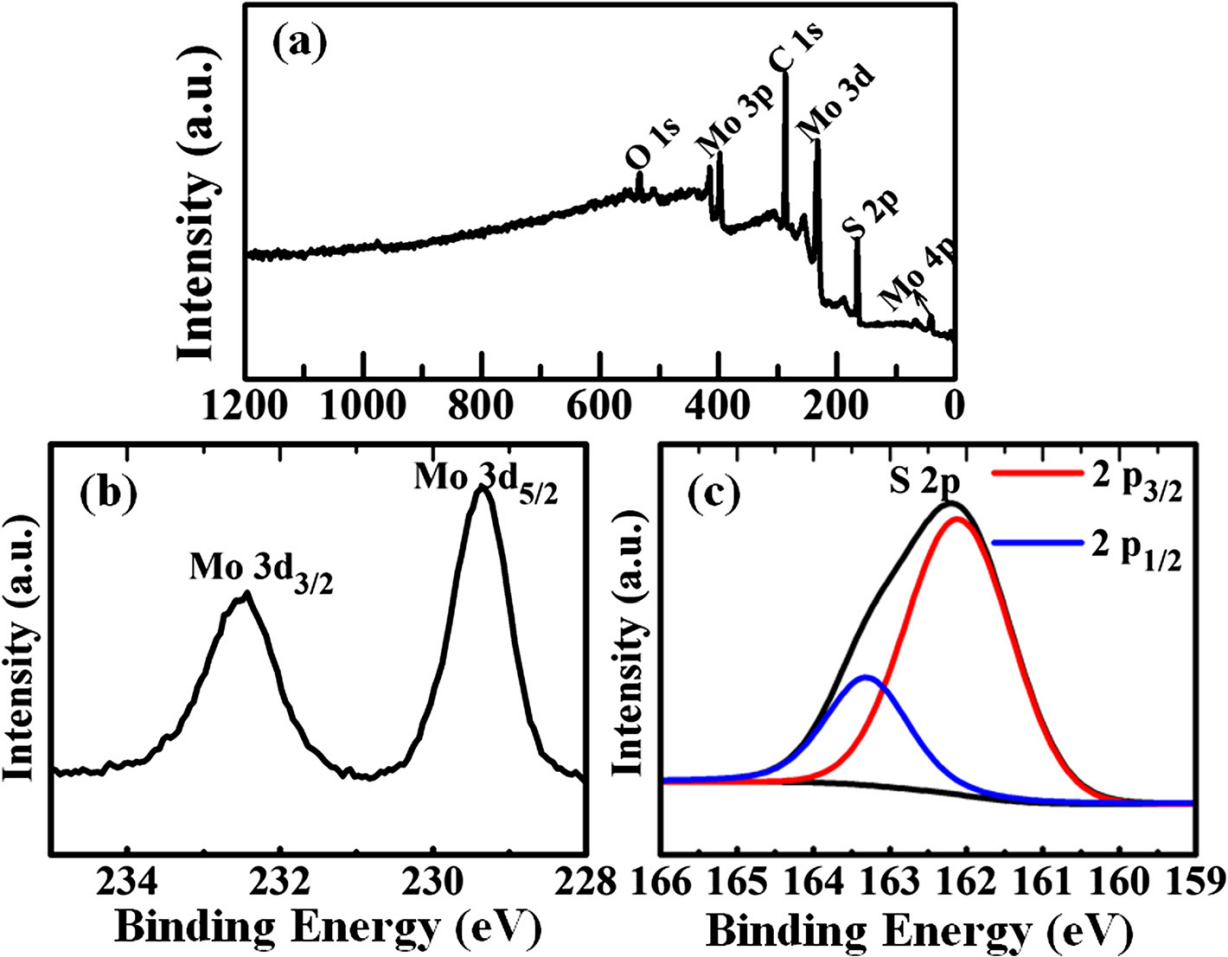
**The XPS results for the MoS**_**2 **_**nanosheets synthesized at 240°C. (a)** The survey spectrum, high resolution scan of **(b)** Mo 3*d* and **(c)** S 2*p.*

The bonding characteristics and the composition of the MoS_2_ nanosheets were captured by XPS, and the representative results are shown in Figure 3a. Besides element of C, the wide XPS spectra of the MoS_2_ nanosheets (240°C) show only signals arising from elements Mo and S. Note that the appearance of some O element may be caused by the surface-adsorbed CO_2_ and O_2_. The Mo 3*d* XPS spectrum of MoS_2_ nanosheets in Figure 3b shows two strong peaks at 229.3 and 232.5 eV, attributed to the doublet Mo 3*d*_5/2_ and Mo 3*d*_3/2_. The peaks, corresponding to the S 2*p*_1/2_ and S 2*p*_3/2_ orbital of divalent sulfide ions (S^2−^), are observed at 163.3 and 162 eV, as shown in Figure 3c. All these results are consistent with the reported values for MoS_2_ crystal [11].

**Figure 4:**
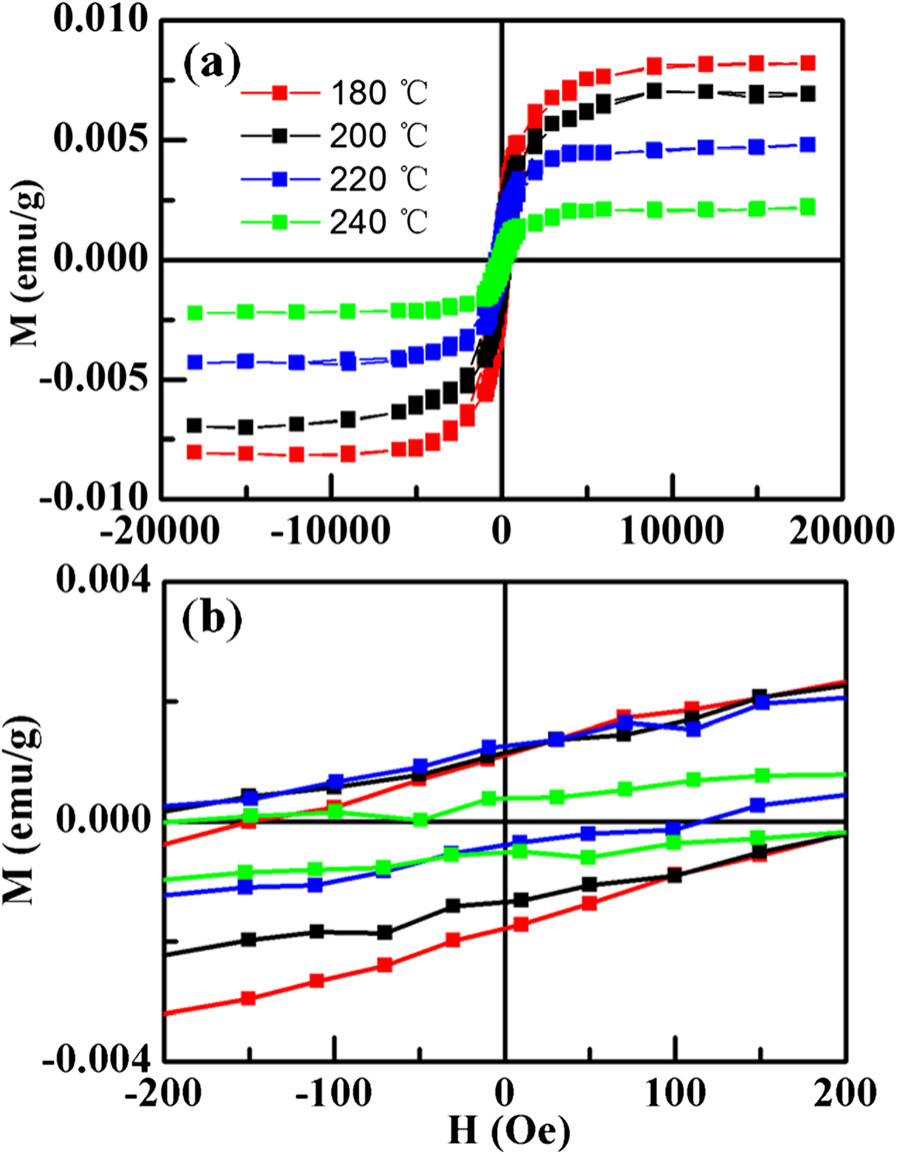
**M-H curves for the MoS**_**2 **_**nanosheets. (a)** M-H curves for the MoS_2_ nanosheets synthesized at different temperatures, **(b)** the zoom of the low field region of the M-H curves for the samples.

**Figure 5:**
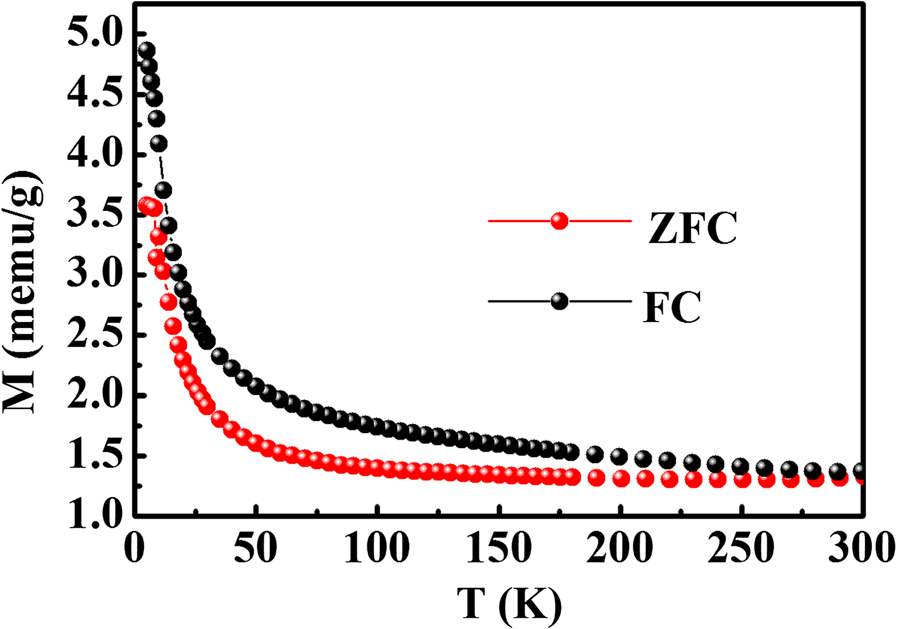
**ZFC and FC curves at the dc field of 100 Oe for the MoS**_**2 **_**nanosheets synthesized at 180°C.**

Figure 4a shows the magnetization versus magnetic field (M-H) curves for the synthesized MoS_2_ nanosheets, where the diamagnetic (DM) signal in the high-field region has been deducted. As can be seen form Figure 4b that all samples show clear coercivity and hysteresis, revealing their room temperature ferromagnetism nature. Results indicate that the ferromagnetism decrease gradually as the reaction temperature increases, and the measured saturation magnetization (*M*_s_) for samples are 0.0073 (180°C), 0.0068 (200°C), 0.0046 (220°C), and 0.0021 emu/g (240°C), respectively. Zero-field-cooled (ZFC) and field-cooled (FC) measures are performed on the representative sample which has the max *M*_s_, and the results are shown in Figure 5. The first measurement was taken after ZFC to the lowest temperature possible (2 K), and in the second run, the measurements were taken under FC conditions. When cooling down from 300 K, both the ZFC and FC data follow similar trend, that is, slow increase of susceptibility until 40 K followed by a sharp rise. Note that the two curves are separated in the whole measured temperature ranges, revealing that the Curie temperature of the sample is expected to exceed 300 K. In addition, the ZFC curve shows no blocking temperature in the range of 2 to 300 K, indicating that there is no ferromagnetic clusters in the sample [23].

Inductively coupled plasma atomic (ICP) results indicate that the contents of the magnetic elements in our MoS_2_ nanosheets (shown in Table 1) are less than 5 ppm. The low contents have been confirmed to have no influence on the observed FM of the samples. Therefore, it is suggested that the observed ferromagnetism here in ultrathin MoS_2_ nanosheets is intrinsic. Recently, similar ferromagnetic nature was also observed in other layered materials like exfoliated WS_2_ nanosheets [26], where the magnetism is considered arising from the unsaturated central metal atom due to partially filled *d* orbitals. In our case, the amorphous MoS_2_ nanosheets may have more defect centers and edges, which would be associated with the undercoordinated Mo atoms, resulting in partially filled *d* orbitals. The high concentration of such edges and defects in our samples could be one of the possible reasons for the observation of ferromagnetism.

**Table 1:** **ICP results for the MoS**_**2 **_**nanosheets**

**Element content (ppm)**	**Fe**	**Co**	**Ni**	**Mn**	**Cr**
First	2.8	1.8	0.8	0.2	1.4
Second	2.5	1.8	0.9	0.3	1.5

## Conclusions

The ultrathin MoS_2_ nanosheets with different crystalline order were fabricated, and their magnetic properties were studied. Results indicate that all the fabricated samples show clear room ferromagnetism and the amorphous sample has larger saturation magnetization owing to having more disordered grain boundary or defects. This unusual ferromagnetism may open perspectives for its application in spintronics devices.

## Competing interests

The authors declare that they have no competing interests.

## Authors' contributions

RZ carried out the synthesis and characterization of the samples, analyzed the results, and wrote the first draft of the manuscript. GD participated in the design, preparation, and discussion of this study. YL contributed ideas for the growth of the samples and revised the manuscript. JQ supervised the research. All authors read and approved the final manuscript.
